# Crystal Structure of a Tetrameric Type II β-Carbonic Anhydrase from the Pathogenic Bacterium *Burkholderia pseudomallei*

**DOI:** 10.3390/molecules25102269

**Published:** 2020-05-12

**Authors:** Andrea Angeli, Marta Ferraroni, Mariana Pinteala, Stelian S. Maier, Bogdan C. Simionescu, Fabrizio Carta, Sonia Del Prete, Clemente Capasso, Claudiu T. Supuran

**Affiliations:** 1 NEUROFARBA Department, Sezione di Scienze Farmaceutiche, Via Ugo Schiff 6, Università degli Studi di Firenze, 50019 Sesto Fiorentino (Florence), Italy; andrea.angeli@unifi.it (A.A.); fabrizio.carta@unifi.it (F.C.); 2 Centre of Advanced Research in Bionanoconjugates and Biopolymers Department, “Petru Poni” Institute of Macromolecular Chemistry, 700487 Iasi, Romania; pinteala@icmpp.ro (M.P.); smaier@ch.tuiasi.ro (S.S.M.); bcsimion@icmpp.ro (B.C.S.); 3 Department of Chemistry “Ugo Schiff”, Via della Lastruccia 13, Università degli Studi di Firenze, I-50019 Sesto Fiorentino (Florence), Italy; 4 Polymers Research Center, Polymeric Release Systems Research Group, “Gheorghe Asachi” Technical University of Iasi, 700487 Iasi, Romania; 5 Istituto di Bioscienze e Biorisorse, CNR, Via Pietro Castellino 111, 80131 Napoli, Italy; sonia.delprete@unifi.it (S.D.P.); clemente.capasso@ibbr.cnr.it (C.C.)

**Keywords:** β-Carbonic Anhydrase, *Burkholderia pseudomallei*, crystal structure, type II CA

## Abstract

Carbonic anhydrase (CA) is a zinc enzyme that catalyzes the reversible conversion of carbon dioxide to bicarbonate and proton. Currently, CA inhibitors are widely used as antiglaucoma, anticancer, and anti-obesity drugs and for the treatment of neurological disorders. Recently, the potential use of CA inhibitors to fight infections caused by protozoa, fungi, and bacteria has emerged as a new research line. In this article, the X-ray crystal structure of β-CA from *Burkholderia pseudomallei* was reported. The X-ray crystal structure of this new enzyme was solved at 2.7 Å resolution, revealing a tetrameric type II β-CA with a “closed” active site in which the zinc is tetrahedrally coordinated to Cys46, Asp48, His102, and Cys105. *B. pseudomallei* is known to encode at least two CAs, a β-CA, and a γ-CA. These proteins, playing a pivotal role in its life cycle and pathogenicity, offer a novel therapeutic opportunity to obtain antibiotics with a different mechanism of action. Furthermore, the new structure can provide a clear view of the β-CA mechanism of action and the possibility to find selective inhibitors for this class of CAs.

## 1. Introduction

*Burkholderia pseudomallei* is the etiologic agent of a severe and often fatal syndrome known as melioidosis, or Whitmore’s disease [1]. Melioidosis is a severe disease of humans and animals, causing an estimated 165,000 cases per year, resulting in a predicted 89,000 deaths [2,3]. Infection with *B. pseudomallei* was usually associated with environmental exposure and can occur through breaks in the skin, inhalation, or ingestion [4]. In addition, *B. pseudomallei* is one of the prominent opportunistic pathogens classified as a bioterrorism agent by both the UK government and the US Centers for Disease Control and Prevention [4,5]. Finally, in recent years, the tolerance to antimicrobials has increased considerably [6,7].

In this scenario, a novel and promising approach for fighting antibiotic resistance is represented by the inhibition of carbonic anhydrases (CAs, EC 4.2.1.1 [8,9,10,11,12], a superfamily of metalloenzymes which catalyzes the simple but physiologically crucial reaction of carbon dioxide hydration to bicarbonate and protons [13,14,15]. These enzymes are present in all life kingdoms and, to date, are divided into eight distinct classes which exhibit no significant sequence or structural similarities, known as the α, β, δ, γ, ζ, θ, η, and the recently discovered ι [16,17]. All the catalytically active CAs contain, independently of the genetic groups, a metal ion cofactor, which is necessary for enzyme catalysis [13,14,15,16,17]. The α-, β-, δ-, γ-CAs use the Zn^2+^ ion as a catalytic metal, in addition, γ-CAs use Fe^2+^ or Co^2+^ ions too [13,14,15]. ζ-CA is cambialistic enzymes, which are active with Cd^2+^ or Zn^2+^ [15,16]. Unexpectedly, the last identified ι-CA, which is encoded in the genome of the marine diatom, *Thalassiosira pseudonana*, prefers Mn^2+^ to Zn^2+^ as a cofactor [17,18]. In addition, in many bacteria, these enzymes are known to be essential for their life cycle, whereas several essential metabolic pathways require either CO_2_ or bicarbonate as a substrate [19,20]. It was demonstrated in vivo that the bacterial growth at an ambient CO_2_ concentration was dependent on CA activity for several species.

The genome of *B. pseudomallei* encodes for β- and γ-CAs. Recently, a gene encoding for the ι-CA was found in the genome of another genus of Burkholderia (*Burkholderia territorii*) [18]. However, neither of the two species had genes encoding for the α-class [18]. This feature is of great interest, because these three classes are not expressed in humans, giving the opportunity to inhibit these classes preferentially. Our group recently reported the catalytic activity and the sulfonamide and anion inhibition profiles of the recombinant β- and γ-CAs from *B. pseudomallei*, named BpsβCA and BpsγCA, respectively [21,22,23]. In the last ten years, numerous results concerning the inhibition profile of the three bacterial CA classes (α, β, and γ) were reported using anions and sulfonamides. Most of these studies were carried out on bacterial CAs from pathogenic bacteria, such as *Francisella tularensis*, *Burkholderia pseudomallei*, *Vibrio cholerae*, *Streptococcus mutans*, *Porphyromonas gingivalis*, *Legionella pneumophila*, *Clostridium perfringens*, and *Mycobacterium tuberculosis* [20,21,24,25,26]. The results indicated that certain CA inhibitors were able to highly inhibit most of the CAs identified in the genome of the aforementioned bacteria. Moreover, certain CA inhibitors, such as acetazolamide and methazolamide, were shown to effectively inhibit bacterial growth in cell cultures [27].

Here, we reported for the first time the crystallographic structure of BpsβCA that was solved in order to understand its function, and laid down the foundation for developing inhibitors that were more potent and selective towards this isoform.

Previous works on the β-CAs class revealed two distinct subtypes of this enzyme called type I or type II β-Cas, according to their active-site organization. [28] Type I presents in the active site the zinc ion coordinated with one histidine, two cysteine residues, and a fourth coordination site occupied by water or a substrate analogue (the so-called open conformation). This particular conformation was reported for the β-CAs from the bacteria, such as *Pisum sativum* [29], *Methanobacterium thermoautotrophicum* [30] and *M. tuberculosis* (Rv1284) [28]. On the other hand, the Type II subclass of β-CAs has a unique zinc-coordination geometry, in which the water molecule is replaced by an aspartate side chain, forming a non-canonical CA active site (the closed conformation), as observed in *Haemophilus influenza* [31,32], *Escherichia coli* [33], *Porphyridium purpureum* [34], and *M. tuberculosis* (Rv3588c) [28]. This subtype is characterized by little or no CO_2_ hydration activity at pH values less than 8.0. Therefore, it was hypothesized that the closed conformation (called T state) observed in the structures of type II β-CAs is an allosteric form of the enzyme and, is the inactive form at pH values below 8.0. However, at pH values larger than 8.3, the closed active site is converted to an open one, with an incoming water molecule replacing the carboxylate moiety of the Asp residue, thus generating the nucleophile required in the catalytic cycle. This was demonstrated by X-ray crystallography (and kinetic studies) in an elegant work by Jones and coworkers [28]. Indeed, at this pH value, the carboxylate of the Asp has a strong interaction with the guanidine/guanidinium moiety of a conserved Arg residue present in all β-CAs investigated so far [28].

## 2. Results

First of all, the catalytic efficiency of recombinant BpsβCA for the physiologic reaction, CO_2_ hydration to bicarbonate and protons, was measured and its kinetic parameters were compared with those of γ-CA and ι-CA classes, CAs from the same gram-negative genus (Table 1).

Data of Table 1 shows similar activities among the different classes of CAs from the Burkholderia genus, possessing a moderate but significant CO_2_ hydrase activity with kinetic parameters (k_cat_) spanning between 1.6 to 5.3 × 10^5^. Furthermore, the activity of BpsβCA is only moderately inhibited (K_i_ of 745 nM) by the clinically used sulfonamide inhibitor acetazolamide (5-acetamido-1,3,4-thiadiazole-2-sulfonamide), which was a much better inhibitor of the other two enzymes belonging to different classes [18,21,22].

**Figure 1:**
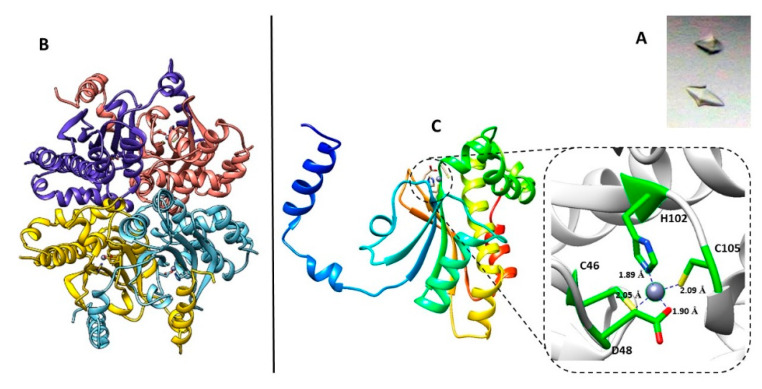
(**A**) The shape of BpsβCA crystals, under bright field illumination. (**B**) Ribbon diagram showing the tetrameric arrangement of BpsβCA. (**C**) Crystal structure of BpsβCA. Ribbon diagram of the BpsβCA structure, asymmetric unit content, and active site of BpsβCA. The detailing insert shows the enzyme active site with the zinc ion (gray sphere) and its ligands (Cys46, His102, Cys105, and Asp48).

Then, the crystal structure of the recombinant type II β-CA from *B. pseudomallei* was determined at a resolution of 3.1 Å (Table 2). Enzyme crystals were obtained by the sitting-drop vapor diffusion method. They belong to the space group P6_4_22, with one molecule per asymmetric unit. Among the β-CAs of the known structure, the highest level of sequence homology of Bpsβ-CA was observed with the β-CAs from *Pseudomonas aeruginosa* (57.4% identity), *Porphyridium purpureum* (55.3% identity), *Salmonella typhimurium* (49.5% identity) and, finally, from *Vibrio cholerae* (49.3% identity). The structure was solved by molecular replacement using the β-CA from *Pseudomonas aeruginosa* (PsCA3, 57.4% sequence identity, PDB code: 4rxy) as the initial model [36]. The biological assembly was investigated by the PISA (Protein Interfaces, Surfaces and Assemblies) software application, that confirmed a tetrameric organization of the enzyme which strictly resembles that of the other structurally characterized β–CAs, which have a dimer, a tetramer, or an octamer arrangement (Figure 1). The active site is located in a cleft at the interface of one dimer, and contains a zinc ion at the bottom, coordinated by three protein residues, namely Cys46, His102, and Cys105. Furthermore, Asp48 is visible in the fourth coordination position instead of the typical water molecule (Figure 1), revealing a “closed” configuration of the active site. Therefore, the enzyme can be classified as a type II β-CA that, as expected, assumes a “closed” conformation, considering the pH of the crystallization condition (pH 7.5).

**Figure 2:**
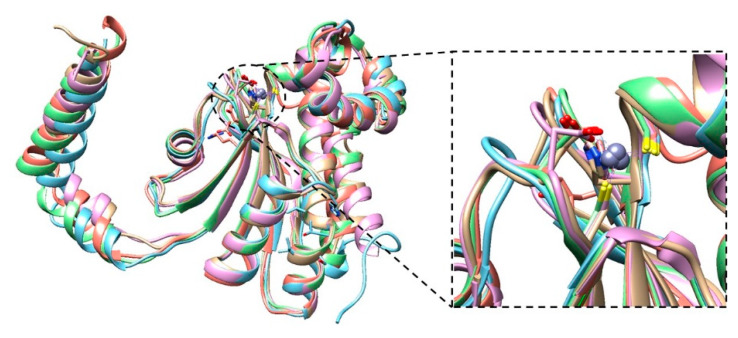
Superposition of the BpsβCA structure (brown), with the previously determined type II β-CAs from *Pseudomonas aeruginosa* (cyan, *r.m.s.d* of 0.784 Å), *Porphyridium purpureum* (violet *r.m.s.d* of 0.749 Å), *Salmonella typhimurium* (green *r.m.s.d* of 0.785 Å), and *Vibrio cholerae* (red *r.m.s.d* of 0.952 Å). The gray sphere represents the zinc atom in the active site. The right panel highlights the active site of type II β-CAs.

The structural comparison of BpsβCA with other β-CAs belonging to different bacterial species, shows substantial conservation of the BpsβCA three-dimensional structure (Figure 2), which is the highest with the *Pseudomonas aeruginosa* (PsCA3).

**Figure 3:**
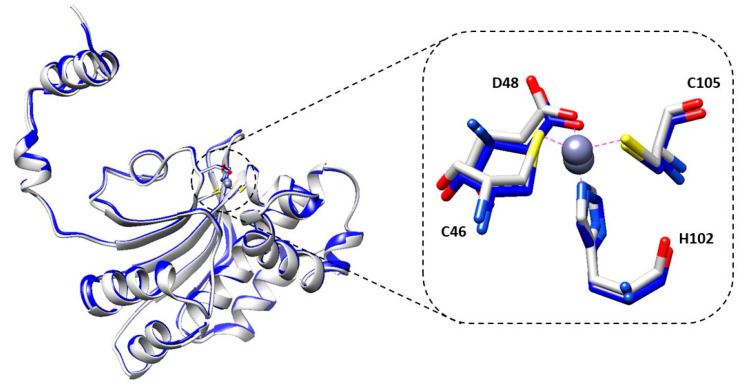
A comparison of the BpsβCA active site arrangements in two different pH conditions: 7.5 and 8.5. In gray: BpsβCA crystalized at pH 7.5. In blue: BpsβCA crystalized at pH 8.5.

The active site is also well conserved compared to the other type II β-CAs. Arg50 is supposed to interact through two hydrogen bonds with Asp48 in the “open” conformation, as inferred from the structures showing the active site of the type I β-CAs. Unfortunately, the electron density maps did not show any density at the side chain of Arg50 that was not included in the model. It was hypothesized that a Tyrosine was necessary for efficient proton transfer inside the mechanism of the β-CAs, for example Tyr212 in *Arabidopsis Thaliana* and Tyr83 in *Vibrio cholerae* β-CA. BpsβCA has phenylalanine (Phe87) at that position and other residues in the active site may play an essential role for catalysis. Finally, to gain information on the open conformation of the enzyme active site, we solved the structure of the enzyme from one crystal obtained in the same crystallization condition, except pH was increased to 8.5, a value at which the enzyme was shown to possess catalytic activity. Although the structure was determined at quite a low resolution (maximum resolution 2.7 Å), we could observe that in the active site, the Asp48 is in the same position as in the structure at pH 7.5, interacting directly with the zinc ion as reported above (see Figure 3).

Nevertheless, the active form was never observed in a β-CA structure, regardless of the crystallization pH, except for in two mutants of *H. influenzae* CAs [26] and a thiocyanate inhibitor complex of *M. tuberculosis* CA [28].

## 3. Materials and Methods

### 3.1. Enzyme Preparation

The identification of the gene encoding for *B. pseudomallei* β-CA (BpsβCA) was performed, as described by Del Prete et al. [22] Briefly, The β-CA gene of *B. pseudomallei* (accession number: WP_004189176.1) was identified by running the Basic Local Alignment Search Tool (BLAST) software application, using the nucleotide sequences of bacterial β-CAs as a query sequence. The GeneArt Company (Invitrogen), specializing in gene synthesis, designed the synthetic BpsβCA gene (BpsβCA-DNA) encoding for the BpsβCA (a protein made of 256 amino acid residues) containing four base-pair sequences (CACC) necessary for directional cloning at the 50 end of the PfCAdom gene. The recovered BpsβCA gene and the linearized expression vector (pET-100/D-TOPO) were ligated by T4 DNA ligase to form the expression vector pET-100/BpsβCA. BL21 DE3 codon plus competent cells (Agilent) were transformed with pET-100/BpsβCA, grown at 37 °C, and induced with 1 mM IPTG. After 30 min, ZnSO_4_ (0.5 mM) was added to the culture medium (2 L), and cells were grown for additional 3 h. Subsequently, cells were harvested and resuspended in the following buffer: 50 mM Tris/HCl, pH 8.0, 0.5 mM PMSF, and 1 mM benzamidine. Cells were then disrupted by sonication at 4 °C. After centrifugation at 12,000× *g* for 45 min, the supernatant was incubated with His Select HF nickel affinity gel resin (Sigma) equilibrated in lysis buffer for 30 min. Following centrifugation at 2000 g, the resin was washed in wash buffer (50 mM Tris/HCl, pH 8.3, 500 mM KCl, and 20 mM imidazole). The protein was eluted with the wash buffer containing 300 mM imidazole. Collected fractions were dialyzed against 50 mM Tris/HCl, pH 8.3. At this stage of purification, the protein was at least 95% pure, and the obtained recovery was about 20 mg of the recombinant protein.

### 3.2. Crystallization and Data Collection

The enzyme was crystallized at 296 K using the sitting-drop vapor-diffusion method in 96-well plates (CrystalQuick, Greiner Bio-One, Maulbronn, Germany). Drops were prepared using 1 µL of protein solution mixed with 1 µL of reservoir solution and were equilibrated against 100 µL precipitant solution. The concentration of the protein was 10 mg mL^−1^ in 50 mM Tris pH 7.2. Initial crystallization condition was found using the JCSG plus screen kit (Molecular Dimensions) and were optimized. Diffraction-quality crystals grew within four months from a solution consisting of 22% PEG 4000, 10% isopropanol, 100 mM HEPES pH 7.5 or 8.5, and 3% *v/v* 1,5-Diaminopentene di-HCl. The crystals belonged to the primitive hexagonal space group P6422. Data obtained from crystals at pH 8.5 were collected on the XRD2 beamline at Elettra, Trieste, Italy, using a Pilatus3_6M Dectris CCD detector and a wavelength of 1.000 Å. Data obtained from crystals at pH 7.5 was collected on the ID-29 beamline at ESRF (Grenoble, France) with a wavelength of 1.0399 Å and a Pilatus3_6M Dectris CCD detector. For data collection, a crystal of the enzyme was cooled to 100 K using a solution consisting of 22% PEG 4000, 10% isopropanol, 100 mM HEPES pH 7.5 or 8.5, 3% *v/v* 1,5-Diaminopentene di-HCl, and 15% ethylene glycol, as cryoprotectant. The data were processed with an XDS program package [37].

### 3.3. Structure Determination and Refinement

The structure was solved by the molecular-replacement technique using the MOLREP program for molecular replacement [35] using the coordinates of the structure of β-carbonic anhydrase from *P. aeruginosa* (PDB entry 4rxy) as a starting model. The model was refined using the REFMAC5 program [38] from the CCP4 suite [39]. Manual rebuilding of the model was performed using the Crystallographic Object-Oriented Toolkit (Coot) [40]. Solvent molecules were introduced automatically using the ARP/wARP software suite [41]. Data processing and refinement statistics are summarized in Table 1. Protein coordinates were deposited in the Protein Data Bank (PDB entry 6YL7; 6YJN). Structural figures were generated with the UCSF Chimera package [42].

### 3.4. Kinetic and Inhibition Assay

An Applied Photophysics stopped-flow instrument was used for assaying the CA catalyzed CO_2_ hydration activity. [43] Phenol red (at a concentration of 0.2 mM) was used as an indicator, working at the absorbance maximum of 557 nm, with 20 mM TRIS (pH 8.3) as buffer, and 20 mM NaClO_4_ (for maintaining constant the ionic strength), following the initial rates of the CA-catalyzed CO_2_ hydration reaction for a period of 10–100 s. The CO_2_ concentrations ranged from 1.7 to 17 mM for the determination of the kinetic parameters (by Lineweaver-Burk plots) and inhibition constants. For each inhibitor, at least six traces of the initial 5–10% of the reaction were used for determining the initial velocity. The uncatalyzed rates were determined in the same manner and subtracted from the total observed rates. Stock solutions of the inhibitor (10–100 mM) were prepared in distilled-deionized water and dilutions up to 0.01 mM were done thereafter with the assay buffer. Inhibitor and enzyme solutions were preincubated together for 15 min at room temperature prior to assay, in order to allow for the formation of the E-I complex, or for the eventual active site mediated hydrolysis of the inhibitor. The inhibition constants were obtained by non-linear least-squares methods using PRISM 3 and the Cheng–Prusoff equation, as reported earlier, and represent the mean from at least three different determinations. All CA isoforms were recombinant ones obtained in-house. All salts and small molecules were of the highest purity available, from Sigma-Aldrich (Milan, Italy).

## 4. Conclusions

The X-ray crystal data of the recombinant β-CA from *Burkholderia pseudomallei* (BpsβCA) are reported in this paper. The X-ray crystal structure of the enzyme was solved at 2.7 Å resolution and two different pH levels (7.5 and 8.5). BpsβCA was revealed to be a tetrameric type II β-CA with a closed active site in which the zinc is tetrahedrally coordinated to Cys46, Asp48, His102, and Cys105. The X-ray structure solved at two pH levels (7.5 and 8.5) showed the same “close” conformation at the active site. The genome of *B. pseudomallei* encodes for different classes of CAs (β and γ). Besides, the bacterial CAs play a pivotal role in the life cycle and pathogenicity of the microorganism, balancing their endogenous equilibrium between CO_2_ and HCO_3_^−^. The resolution of the BpsβCA structure provides new insights for the understanding of the enzyme catalytic site, as well as the possibility of finding selective inhibitors for β-CAs. These findings offer the opportunity to obtain new antibiotics that are able to impair the growth or the virulence of the microorganism, with a mechanism of action different to that of the existing drugs.

## Figures and Tables

**Table 1:** Kinetic parameters for the CO_2_ hydration reaction catalyzed by the β- and γ-CAs from *B. pseudomallei* and ι-CA from *B. territorii* measured at 20 °C, pH 8.3 in 20 mM TRIS buffer, and 20 mM NaClO_4_ [20,21,35]. Acetazolamide inhibition data are also shown.

Enzyme	Activity Level	Class	k_cat_ (s^−1^)	k_cat_/K_m_ (M^−1^ s^−1^)	K_i_ (Acetazolamide) (nM)
BpsβCA	Moderate	β	1.6 × 10^5^	3.4 × 10^7^	745
BpsγCA	Moderate	γ	5.3 × 10^5^	2.5 × 10^7^	149
BteCAι	Moderate	ι	3.0 × 10^5^	9.7 × 10^7^	64.9

**Table 2:** Summary of Data Collection and Atomic Model Refinement Statistics.

	BpsβCA pH 7.5	BpsβCA pH 8.5
PDB ID	6YL7	6YJN
Wavelength (Å)	1.0399	1.0000
Space Group	P6_4_22	P6_4_22
Unit cell (a, b, c, α, β, γ) (Å,°)	88.74;88.74;112.43;	88.03;88.03;111.64;
	90.0;90.0;120.0	90.00;90.00;120.00
Limiting resolution (Å)	45.37–3.16 (3.38–3.16)	45.04–2.70 (2.83–2.70)
Unique reflections	4858 (743)	13330 (2141)
Rmerge (%)	27.4 (265.4)	26.0 (789.6)
Rmeas (%)	28.08 (271.5)	26.7 (811.5)
Redundancy	24.7 (23.4)	18.4 (18.6)
Completeness overall (%)	99.7 (98.7)	99.9 (99.6)
<I/σ(I)>	10.42 (1.15)	10.93 (0.34)
CC (1/2)	99.8 (59.4)	99.9 (32.1)
**Refinement statistics**		
Resolution range (Å)	45.412–3.166	45.080–2.701
Unique reflections, working\free	4605\3440	7508\7093
Rfactor (%)	19.26	21.4
Rfree(%)	29.62	32.4
r.m.s.d. bonds(Å)	0.0060	0.0052
r.m.s.d. angles (°)	1.5874	1.4987
**Ramachandran statistics (%)**		
Most favored	79.3	82.9
Additionally allowed	17.8	11.4
Outlier regions	2.9	5.7
**Average B factor (Å^2^)**		
Solvent	78.811	83.801
